# Experimental Study of a Superabsorbent Polymer Hydrogel in an Alkali Environment and Its Effects on the Mechanical and Shrinkage Properties of Cement Mortars

**DOI:** 10.3390/polym16081158

**Published:** 2024-04-20

**Authors:** Ali Al-Shawafi, Han Zhu, Sadi Ibrahim Haruna, Yasser E. Ibrahim, Jian Yang, Said Mirgan Borito

**Affiliations:** 1 School of Civil Engineering, Tianjin University, Tianjin 300350, China; ali91@tju.edu.cn (A.A.-S.); hanzhu2000@tju.edu.cn (H.Z.); jianyang1895@tju.edu.cn (J.Y.); mirg_said95@tju.edu.cn (S.M.B.); 2 Engineering Management Department, College of Engineering, Prince Sultan University, Riyadh 11586, Saudi Arabia; ymansour@psu.edu.sa

**Keywords:** superabsorbent polymers, sodium silicate, water retaining, mortar, mechanical properties, cracking resistance

## Abstract

As internal curing self-healing agents in concrete repair, the basic properties of superabsorbent polymers (SAPs), such as water absorption and release properties, are generally affected by several factors, including temperature and humidity solution properties and SAP particle size, which regulate the curing effect and the durability of cementitious composites. This study aimed to investigate the water retention capacities of SAPs in an alkaline environment over extended periods by incorporating liquid sodium silicate (SS) into SAP–water mixtures and examining the influence of temperature. The influence of SAP particle size on mortar’s water absorption capacity and mechanical behavior was investigated. Two mixing techniques for SAPs (dry and pre-wetting) were employed to assess the influence of SAP on cement mortars’ slump, mechanical properties, and cracking resistance. Four types of SAPs (SAP-a, SAP-b, SAP-c, and SAP-d), based on the molecular chains and particle size, were mixed with SS to study their water absorption over 30 days. The results showed that SAPs exhibit rapid water absorption within the first 30 min, exceeding 85% before reaching a saturation point, and the chemical and temperature variations in the water significantly affected water absorption and desorption. The filtration results revealed that SAP-d exhibited the slowest water release rate, retaining water for considerably longer than the other three types of SAPs. The mechanical properties of SAP mortar were reduced due to the addition of an SAP and the improved cracking resistance of the cement mortars.

## 1. Introduction

Superabsorbent polymers (SAPs) are cross-linked polyelectrolytes that swell when in contact with water or aqueous solutions, generating a hydrogel. The amount of water an SAP absorbs can reach several hundred times its weight [1,2]. The incorporation of SAPs reduced the shrinkage cracking strains in cementitious materials in [3,4]. The use of SAPs has attracted the attention of many researchers who want to improve the self-healing ability of cementitious materials and reduce the maintenance cost of concrete structures. Self-healing materials are distinguished by an autonomously initiated repair process upon crack formation and offer a solution in the event of limited access. Cementitious materials can seal and repair cracks by autogenous healing, primarily produced by the continuing hydration of unhydrated cement particles and the precipitation of CaCO_3_ [5,6]. While these mechanisms happen concurrently, the further hydration of cement particles occurs only when all unhydrated cement has been consumed. Calcium hydroxide continues to dissolve and carbonate with time [7]. The availability of water inside the concrete fracture is critical for the commencement of these processes and, as a result, autogenic healing. SAPs have been extensively studied in previous studies to preserve humidity inside the crack [8,9,10]. SAPs comprise ionic monomers that allow them to uptake a large fluid capacity by osmotic pressure. Thus, the absorption of SAPs is subsequently dependent on the concentration of ions in the swelling medium. An SAP hydrogel can retain most of its water absorbed under pressure with mechanical integrity because of its type of cross-linking network structure [1]. The cross-linking of an SAP is, therefore, of great importance because it prevents a hydrophilic polymer from dissolving in water, and it provides an SAP network structure that retains most of its absorbed water even after pressing, and the cross-linking also gives the hydrogel mechanical stability. Several studies have been carried out to investigate internal curing and autogenous shrinkage reduction in relation to SAPs in cementitious composites [5,11,12,13]. Polymer materials have exhibited several engineering applications [14,15,16].

Niu et al. [17] studied the effect of SAP particle size on the microstructure and compressive strength of concrete. The study found that larger SAPs result in higher average porosity, effectively reducing overall porosity. The compressive strength of cement paste is negatively affected due to the addition of SAPs. Qin et al. [18] reported that the ionic valence substantially affects SAPs’ water absorption capacity, implying that the bigger the ionic radius, the less the SAP absorbs. SAPs’ water absorption rate increases as the temperature of the solution rises. Snoeck et al. [19] employed nuclear magnetic resonance (NMR) to explore the water release effect of SAPs in cement-based material during hydration, and they established water release kinetics that efficiently reduce autogenous shrinkage. Mehdi et al. [19] investigated the water motion of cement-based material during hydration using neutron radiography. Schröfl et al. [10] and Kang et al. [20] investigated SAPs’ water absorption capacity. They suggested that absorption in a solution of SAP is primarily measured by osmotic pressure, which may be seen as a diffusion process and described by Fick’s second law. The total ion concentration and [Ca^2+^] concentration in the cement hydration process affected the SAPs’ absorption capacity [21,22]. Oh and Choi [23] studied the influence of SAPs on the rheological properties of cement mortars. Their study focused on examining the performance requirements for the 3D printing of cement-based materials. Tan et al. [24] reported that agglomerative accumulation is developed in the mortar void after water desorption from an SAP with sufficient hydration products, such as C-S-H gels, around the SAP voids. The authors of this study investigated the effect of including the SAP in a cement mortar under different dosing approaches.

However, few studies have investigated SAPs’ water absorption and release in cementitious composites. The process of how much water an SAP absorbs and retains in a cementitious environment, like fresh concrete, and, eventually, how it releases all the water when the concrete becomes hardened has not been well understood quantitatively. Such a process is complex to examine experimentally, and it is not practical to break concrete and observe SAP traces every time during concrete hardening. Cement is an alkali material that is similar to sodium silicate (SS). Therefore, this study used an experiment plan which mixed an SAP with water, and then SS was added to the mix, and the water-releasing effect was experimentally observed. In this study, liquid SS was used to create an alkali environment, and two SAP mixing techniques (dry and pre-wetting) were utilized to study the influence of SAP particle size on cement mortars’ slump, mechanical properties, and cracking resistance. Moreover, the SAP mortar’s restrained shrinkage behavior was determined using a restrained shrinkage eccentric ring device. 

## 2. Materials and Methods

### 2.1. Materials

#### 2.1.1. Cement and Aggregate

**Figure 1:**
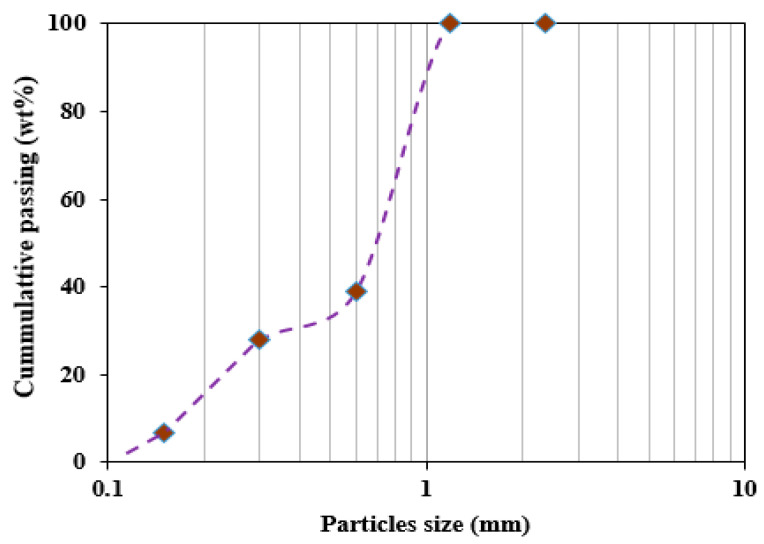
Gradation curve of aggregate used.

OPC Grade 42.5 R was used to produce the mortar modified with SAPs, and the chemical composition of OPC is presented in Table 1. River sand with a fineness modulus of 2.08 and an apparent density of 2670 kg/m^3^ was used as fine aggregate. The sand had a particle size ranging from 0.3 to 2.26 mm; the particle distribution curve of the sand is depicted in Figure 1. The sieve analysis of the river sand was conducted following ASTM C136/C136M-19 [25].

#### 2.1.2. Superabsorbent Polymers (SAPs) and Sodium Silicate (SS)

**Figure 2:**
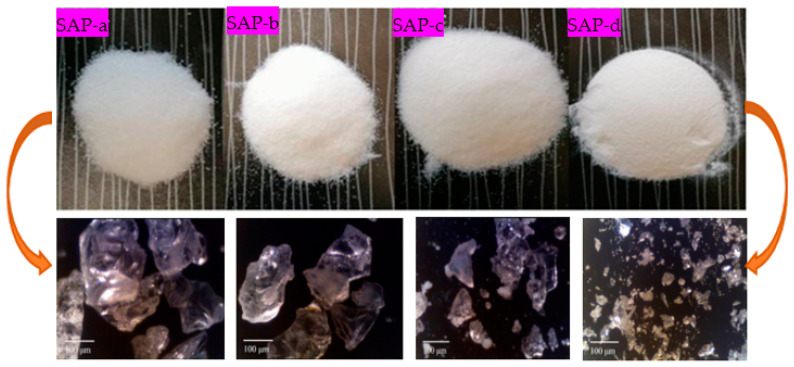
Microscopic particle size observation of dry superabsorbent polymers.

In this study, four types of SAPs, classified as SAP-a, SAP-b, SAP-c, and SAP-d, were employed based on their different molecular chains and particle sizes to study hydrogels derived from them in an alkali environment and their influence on the cement mortars’ hardening and durability properties. Figure 2 shows the microscopic particle sizes and surface textures of the four different SAPs used in this study, while the physical properties of the SAPs are summarized in Table 2.

The sodium silicate liquid we used is an aqueous solution produced by Wuxi, Yatai, United Chemical Co., Ltd., Tianjin Binhai-Zhongguancun Science and Technology Park, China. The chemical composition of sodium silicate is summarized in Table 3. The sodium silicate liquid was used as an alkaline environment to simulate the cementitious-based material. 

#### 2.1.3. Mix Proportion and Specimen Preparation

**Figure 3:**
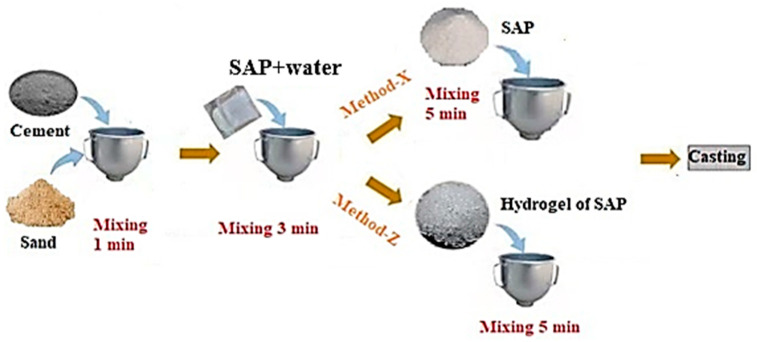
The systematic procedure of preparing cement mortars modified with SAPs derived from Method-X and Method-Z.

Two types of mortar were prepared for this study according to the preparation of the SAP admixture. Each SAP was introduced to the cement mortar based on the two kinds of preparation methods, namely Method-X and Method-Z. Figure 3 presents the procedure adopted for including SAPs in the mortar mixtures, and the mix proportion is summarized in Table 4. To improve the workability of the mortar mixtures, a carboxylate-based superplasticizer was added to the mixture according to the design mix shown in Table 4. 

Method-X involves an SAP absorbing mixed water in a highly active alkali environment. The process involves weighing and combining cement and sand, then thoroughly mixing for 1 min in a mortar bowl. Two-thirds of the water is gradually introduced while mixing at a low speed for another minute. Subsequently, the remaining water portion and the water-reducing agent are slowly introduced to the mixture. Then, the mixtures containing SAPs are blended for 5 min at a high speed. A steel mold is used to cast the samples, and then the samples are covered with plastic to prevent moisture escape. 

Method Z focuses the maximum absorption of SAP particles on mortar mixture. The method is initiated by pre-soaking the prescribed SAP particles in a 500 mL beaker and mixing them thoroughly for 2 min. To avoid water evaporation, the beaker is sealed with a plastic sheet and utilized after it has reached equilibrium. Then, the mortar constituent materials are mixed according to the design mix for 4 min. The SAP gels are gradually added into the dry mixing mortar materials and mixed for 5 min at a high speed. The fresh mortar is cast into a prism mold with dimensions of 40 × 40 × 160 mm^3^. The cast samples are removed from the mold after 254 h. 

### 2.2. Testing Procedure

#### 2.2.1. Water Absorption Capacities of SAPs

SAPs’ water absorption capacities are primarily determined by grain size variations and the cross-linking density [26]. As a result, it is vital to evaluate the water absorption capabilities of various kinds of SAPs in tap water. The filtration process has been widely evaluated as appropriate for using SAPs in cement materials [27,28]. The experiment was conducted in two phases to determine its viability. The first phase focused on evaluating the absorption and desorption characteristics of SAPs under varying temperature conditions using a filtration technique and SS liquid to simulate the cementitious environment. Based on the findings of the initial phase, two types of SAPs with distinct absorption and desorption behaviors were chosen for the second phase. These SAPs were incorporated into mortar matrices at concentrations of 1%, 2%, and 3% of cement weight. The absorption and water retention capacity of the superabsorbent polymer (SAP)–water mixes were evaluated under various temperature conditions. The absorption and water retention capacity were measured at room temperature over 720 h. Additionally, the impact of extreme temperatures, both low (−5 °C) and high (60 °C), was examined for 24 h.

**Figure 4:**
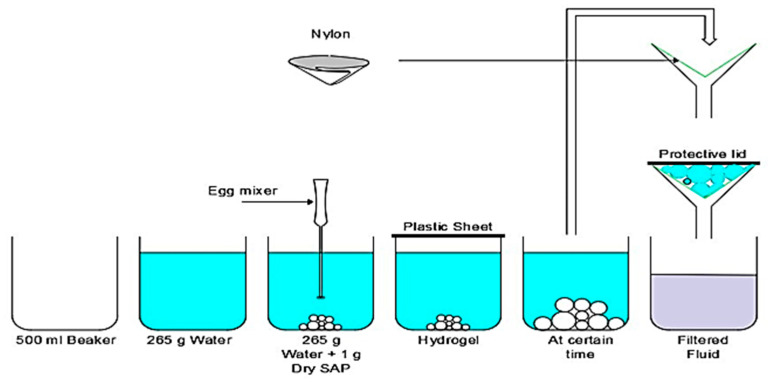
Procedures for SAP–water mixes and filtration method at 25 °C.

At 25 °C, 1 g of SAP was thoroughly mixed with 265 g of tap water using an egg beater (see Figure 4) for 60 s, resulting in a homogeneous gel-like mixture. This mixing process ensured the uniform dispersion of the SAP particles and simulated the mechanical stresses encountered while mixing cementitious materials. The prepared hydrogel was then sealed with a plastic sheet and stored at room temperature until the designated testing times. Absorption capacity measurements were performed at 1, 5, and 30 min, followed by further measurements at 1, 3, 6, 12, 24, 240, 480, and 720 h.

**Figure 5:**
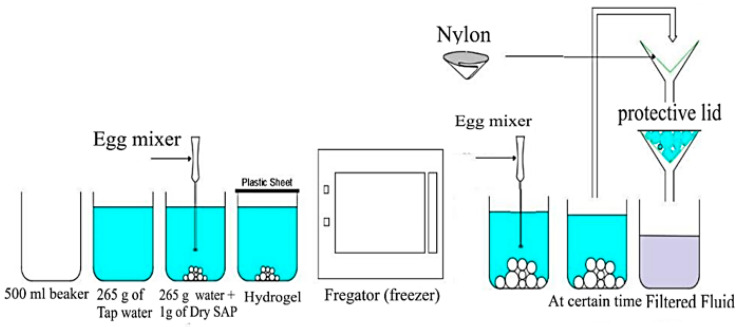
Procedures for SAP–water mixes and filtration method at −5 °C.

Figure 5 shows the procedure of preparing SAPs in a low-temperature (−5 °C) experiment, which involved freezing 265 g of tap water in a culture dish until it reached −5 °C. One gram of dry SAP was then added to the frozen water and vigorously mixed with an egg beater for 60 s. The absorption capacity was promptly measured at 1, 5, and 30 min to capture the initial absorption behavior at low temperatures. To evaluate the long-term absorption rate at −5 °C, the SAP–water mixture was returned to the freezer and remixed every 10 min to prevent freezing. Absorption rate measurements were conducted at 1, 3, 6, 12, and 24 h.

**Figure 6:**
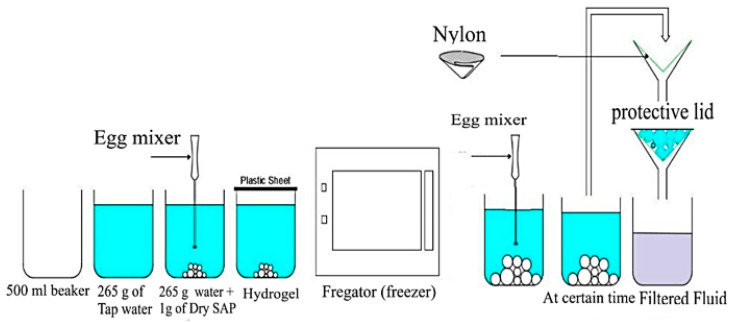
Procedures for SAP–water mixes and filtration method at 60 °C.

In a high-temperature (60 °C) experiment, 265 g of tap water was heated to 60 °C in an oven, and 1 g of dry SAP was then rapidly mixed with hot water for 60 s using an egg beater. The SAP–water mixture was immediately placed back in the oven at 60 °C to maintain the elevated temperature throughout the experiment, as shown in Figure 6. Absorption rate measurements were recorded at 1, 3, 6, 12, and 24 h. All samples were filtered at the specified testing times using an 80 μm nylon paper to determine absorption and water retention capacity accurately. To eliminate any potential influence of suction from the nylon paper, it was first pre-saturated with the test fluid, and its weight was tared before filtration. During filtration, a lid was placed over the filter to prevent evaporation. Filtration was continued until no more liquid droplets fell for one minute. 

#### 2.2.2. Water Retention (WR) Test

The effect of SS was analyzed to study the water retention ability of the hydrogel mixes. Water retention was also used as a cementitious material component. Two commonly used water reducers in mortar were selected: polycarboxylate- and naphthene-type reducers, noted as W.R (L) and W.R (P). The quantities of the W.R. were 2.65 g and 5.5 g. The experimental procedures were as follows: (1) SS addition: The same method of measurements was applied as mentioned in Section 2.2.1. After mixing SAP–water with an egg beater (60 s), the hydrogel was sealed with a plastic sheet for 30 min (the time required for the water absorption of SAPs to form a homogenous mixture of a saturated SAP). Then, SS was added to the hydrogel. The experiment was repeated 12 times for all the SAP types and sealed again with a plastic sheet to avert evaporation until testing time. Finally, the filtration of hydrogel–sodium silicate (SS) mixes was analyzed following the procedures illustrated in Figure 4. 

Filtration was carried out until no drops of water were observed within 1 min. Each filtered fluids’ mass (*m*_3_) was measured. Three specimens were examined for the SAP water absorption test to improve the accuracy of the results. The hydrogels were not reused. The expression for the determination of SAPs’ absorption capacity (AC) at a particular time is expressed in Equation (1). The hydrogel–sodium silicate was mixed with 265 g of water, and the pH of the hydrogel was 6.5 to 7, though after SS addition, it reached 12–13, as observed using litmus paper.

(1) $$AC=\frac{m_{2}-m_{3}}{m_{1}}$$

where *m*_1_ is the dry SAP’s mass, *m*_2_ is the mass of the hydrogel at a particular time, and *m*_3_ is the mass of the fluid. 

#### 2.2.3. Fluidity Test

**Figure 7:**
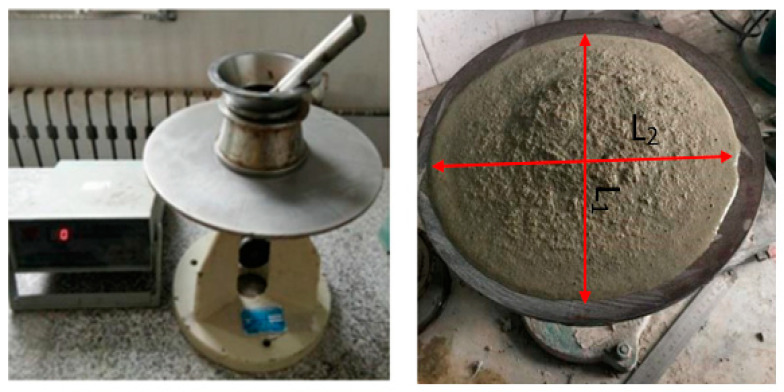
Slump flow test setup.

The fluidity of the cement mortars modified with SAP treated under Method-X and Method-Z was determined following Chinese national GB/T2419-2005 [29]. It is essential to set the mix ratio and reference the consistency of the mortar. During the test, the diameter of the mortar spread was measured after it had been dropped onto a flow table from a height of 12.5 mm and tapped 25 times within 15 s. The test was conducted using an NLD-3 mortar fluidity tester (see Figure 7). The fluidity of the SAP mortars was estimated as the average of the major diameter (*L*_1_) of the spread mortar and the diameter (*L*_2_) of the spread mortar perpendicular to the biggest diameter (*L*_1_), as shown in Equation (2).

(2) $$Slump\;flow\;=\;\left(\frac{L_{1}-L_{2}}{2}\right)$$

#### 2.2.4. Mechanical Properties Test

The mechanical performances of the SAP mortars were determined following GBT 17671-1999 [30] using 30-ton-capacity UTM sets under speed rates of 50 N/s and 2.4 kN/s for the flexural and compressive strength tests, respectively. Sample that were 40 × 40 × 160 mm in size were utilized for the flexural and compressive strength tests and placed on two equal supports with a clear distance of 100 mm. The broken sample from the flexural test was used to determine the compressive strength of the SAP mortar. 

#### 2.2.5. Restrained Shrinkage Properties Test

**Figure 8:**
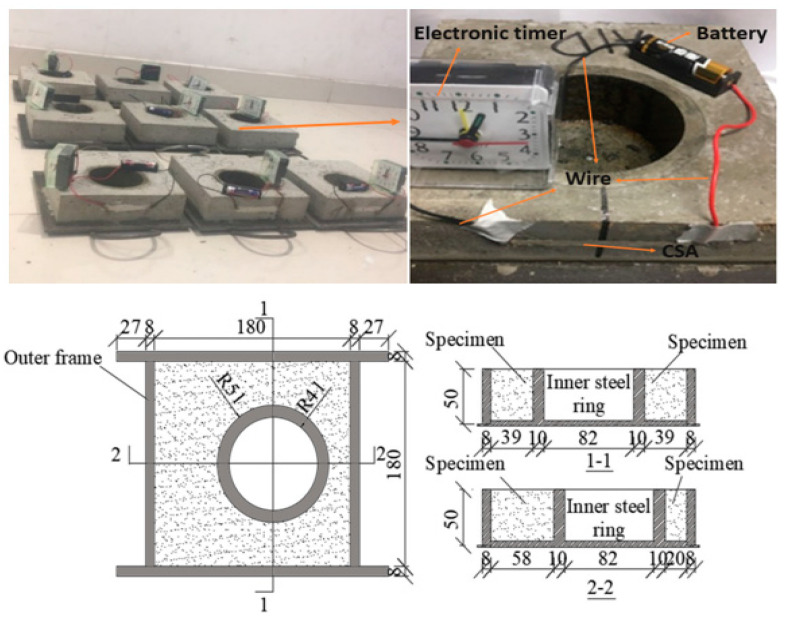
The restrained shrinkage test was set up using an RSERD mold.

The shrinkage behaviors of the SAP mortars were investigated utilizing a restrained shrinkage eccentric ring device (RSERD) mold, as per refs. [13,31,32]. The outline and cross-section of the mold are shown in Figure 8. The mold consists of an exterior frame, an inner steel ring, and a base plate. The RSERD’s inner steel ring is positioned in the center of a external frame, directing crack initiation to the point with the highest strain concentration. For this test, three RSERD specimens were made for each group to investigate the cracking time of SAP mortars containing two SAP types (SAPs-a and SAP-d) for the dry and pre-wetting techniques. The samples were then stored in a room (temperature = 20 ± 2 °C; relative humidity = 50 ± 2%) and cured for 48 h. Paraffin wax was applied to present water evaporation at the specimen surface. 

##### Cracks Observation

An enhanced coating system was used to detect cracking time, commencement, position, and direction [31,32]. The device comprises a simple circuit with an alarm clock, battery, silver paste, conductive paint, etc., as shown in Figure 8. The cracking timing involves the following steps:

- The test pieces were divided into four sides with a rectangular part drawn on the outer side and facing forward.
- We limited our use of conductive adhesive paste (8 cm and 1 cm) because of the stress concentration on the narrow section of specimen in the steel ring.
- Wires were joined to the conductive silver adhesive paste (CSA) application zone and secured with adhesive tape to prevent loosening.
- All the apparatus were connected accordingly to record the cracking time; crack development paths were observed at 5 h intervals.

### 2.3. Analysis of Variance (ANOVA)

In this research, the method of statistical analysis employed was an Analysis of Variance (ANOVA), which was used to assess whether significant disparities existed in the average values across different groups. A three-way ANOVA serves as an effective tool for quantitatively evaluating how the independent variable influences the dependent variables. The significance level for this investigation was established at 95%, corresponding to a *p*-value of 0.05. A *p*-value below 0.05 signifies a statistically significant influence on the dependent variables. For this study, the dependent variables included the types, dosages, mixing methods, and curing time of SAP-modified mortar mixtures. 

## 3. Results and Discussion

### 3.1. Water Absorption Capacities of SAPs

**Figure 9:**
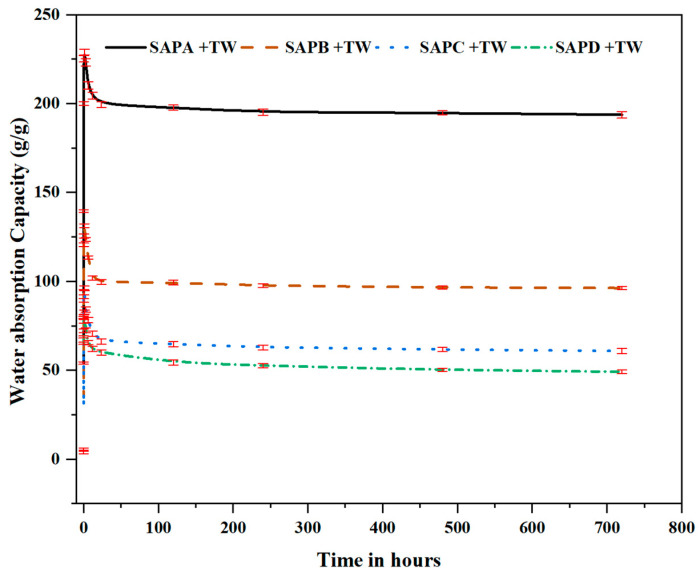
Water absorption capacity rate of SAPs in tap water (TW) for a month.

Figure 9 presents the time-dependent absorption rates of SAPs of varying particle sizes (SAP-a, SAP-b, SAP-c, and SAP-d). All the SAPs exhibit analogous trends in absorption rates, rooted in their standard classification as acid polyacrylic polymers. Notably, the SAPs with coarser particle sizes (SAP-a, SAP-b) exhibited delayed water absorption, while those with shorter particle sizes (SAP-c, SAP-d) demonstrated accelerated rates within the initial 60 min. After this period, the polymer absorbency values revealed distinctive capacities: 229 g/g for SAP-a, 128 g/g for SAP-b, 96.5 g/g for SAP-c, and 82.7 g/g for SAP-d. This result revealed a significant effect of particle size on initial absorption by the SAPs. Subsequently, the water absorption rate gradually decreased until equilibrium, with declines ranging from 13.0% to 0.38% from peak absorption capacity after the initial 24 h. Moreover, between 12 h and 720 h, the decline rate remained below 1%, indicating a relatively stable equilibrium state in the long term. SAP swelling is attributed to the osmotic pressure, linked to the number of ions in the aqueous solution. The polymer network securely holds ions, leading to increased osmotic pressure, and swelling reaches equilibrium when internal and external forces are balanced. The absorption behavior of SAPs is contingent on the concentration of ions in the swelling medium, emphasizing the pivotal role of ion interaction in influencing absorption dynamics. 

The chemical and physical structure behaviors that translate SAP sorption were thoroughly studied by Krafcik et al. [33] utilizing free-radical solution-polymerized poly(acrylic acid-acrylamide)-based SAP compositions containing different dosages of anionic acrylic acid (AA) segments and covalent cross-links in the polymer networks [33,34]. SAP compositions that involved more AA concentrations showed large swelling abilities when immersed in pure water but were very sensitive to the cations in the solution (e.g., Na^+^, K^+^, Ca^2+^, etc.), which resulted in speedy fluid release within minutes after addition in early-age cement filtrate, as reported in [35]. Similarly, Lee at al. [22] suggested that SAPs’ effect on the ion concentration inside cementitious pore fluids can affect the whole hydration process and microstructure development.

### 3.2. The Effect of Temperature on the Water Absorption of the SAPs

The study of the water absorption and retention characteristics of superabsorbent polymers (SAPs) under diverse temperature conditions, such as cold winter (−5 °C), spring/autumn (25 °C), and hot summer (60 °C), provides profound insights into the intricate dynamics of SAP behavior in tap water (TW). The chosen temperature settings reflect the fluctuating temperatures encountered in cement concrete pavement engineering during different construction periods.

**Figure 10:**
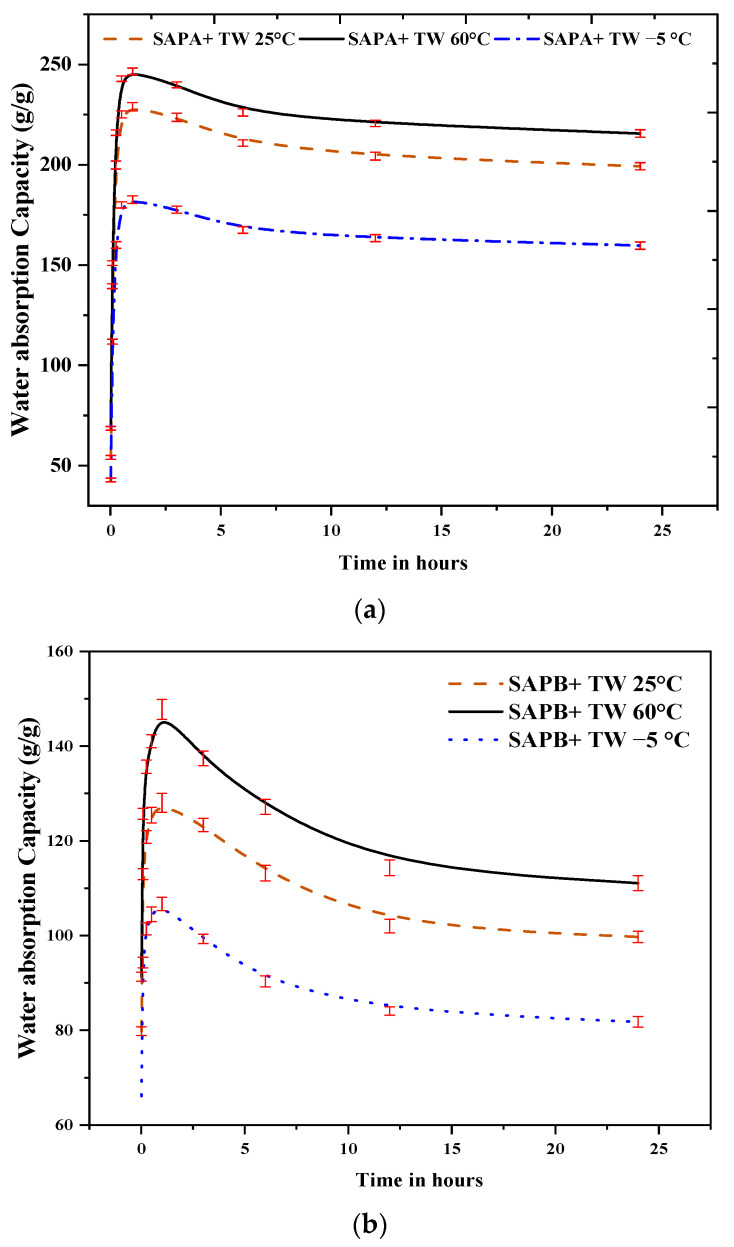

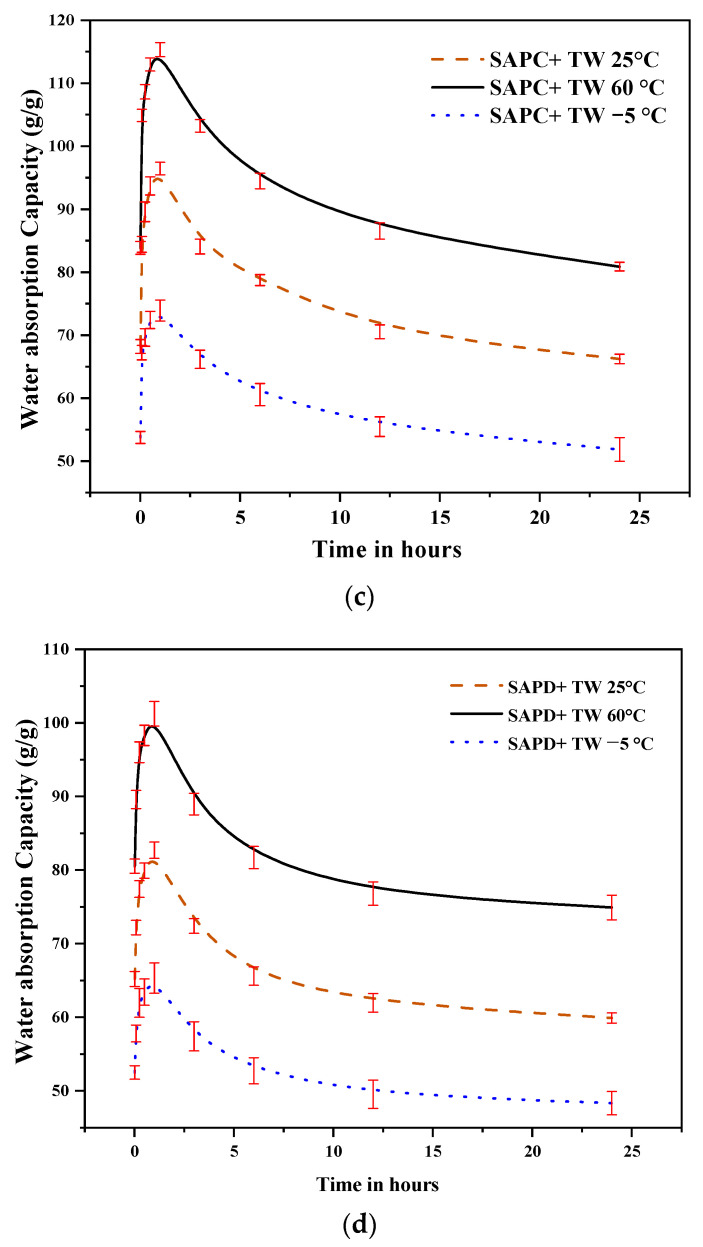
Effect of temperature on the water absorption of different SAPs type treated with tap water for: (**a**) SAP-a, (**b**) SAP-b, (**c**) SAP-c, and (**d**) SAP-d.

Figure 10 shows the results regarding the water absorption capacities of SAPs under different temperature conditions. The results indicated higher temperatures significantly increased the absorption rates and shortened the equilibrium times for SAP gel swelling. This temperature-dependent behavior can be attributed to the activation of SAP polymer chain motion units at elevated temperatures. The heightened motion energy enables these units to overcome energy barriers, leading to various types of motion, including whole chain motion, chain segment motion, and chain joint motion. This activation significantly enhances the diffusion coefficient of the SAP gel network, expediting water absorption. It can be noted from Figure 10 that at 60 °C, peak absorption occurred after 1 h, with water absorption capacities of 246.5, 147.7, 115.3, and 101.2 g/g for SAP-a, SAP-b, SAP-c, and SAP-d, respectively. Conversely, at 25 °C, the water absorption capacities were 229, 128, 96.5, and 82.7 g/g, and at −5 °C, they were 182.6, 106.7, 74, and 65.3 g/g, indicating a clear trend of increased water absorption ability with rising liquid temperatures.

Furthermore, the study of water release and retained capacity under different temperatures and time intervals (Figure 10), simulating SAP behavior in cement paste with the addition of sodium silicate (SS), yielded insightful results. In cold conditions (−5 °C), the water release capacities were notably diminished, with SAP-a exhibiting a reduction of approximately 33.7% compared to 25 °C. In moderate conditions (25 °C), the water release capacities ranged from 82.7 g/g to 229 g/g, showcasing variations among the SAP types. Notably, SAP-a consistently retained the most water, showing 14.1% more water retention than SAP-d at 25 °C. Conversely, at 60 °C, a substantial increase in water release was evident, with SAP-a releasing 113.98 g/g, reflecting an increase of around 20.9% compared to 25 °C.

### 3.3. Water Retention Test

**Figure 11:**
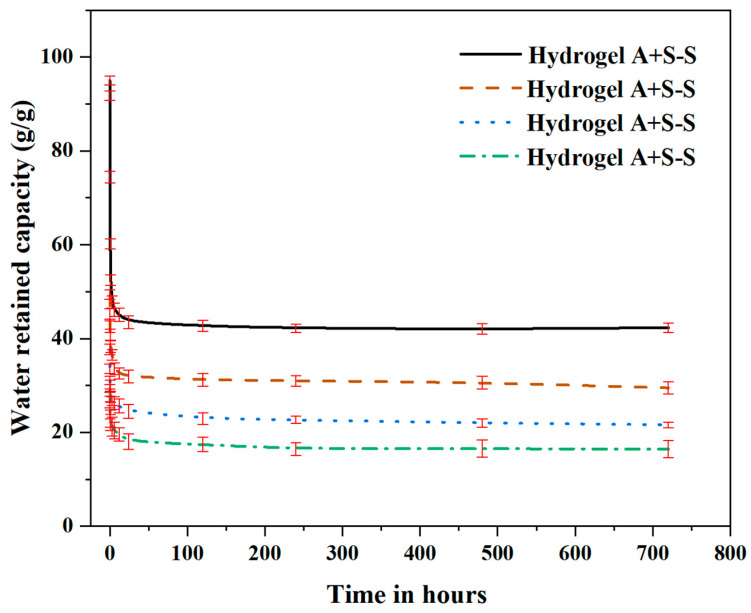
Water retention capacity of SAP hydrogels with SS (observed for a duration of one month).

The behavior of the SAP gels in response to a changing environment revealed an intriguing pattern of water release and retention within a one-month duration, as shown in Figure 11. Introducing sodium silicate (SS) to replicate a cement condition generated an instantaneous release of water by the SAP gels, perhaps due to the variations in particle size and molecular chain order among different the SAP types. For all the SAP types, the SAP gels demonstrated a swift release of water at the initial times until they reached peak capacities. Specifically, SAP-a, which is characterized by having the largest particle size and molecular chain order, had a water retention of 94.98%, followed by SAP-b, SAP-c, and SAP-d, which are characterized by progressively smaller particle sizes and molecular chain orders, exhibiting water retention capacities of 49.35%, 37.68%, and 29.36%, respectively. This pattern featured the influence of particle size and molecular chain order on the dynamics of initial water release. Moreover, the SAP gels stabilized within 720 h, indicating a gradual decrease in water retention capacities. Despite its initial dominance, SAP-a exhibited a 55.06% reduction and stabilized at 42.3%. A decreasing trend was also noted in SAP-b, SAP-c, and SAP-d, with reductions of 29.8%, 45.43%, and 43.89%, respectively, and these gels stabilized at 29.48%, 21.63%, and 16.44%. These percentage reductions describe the prolonged water retention capacities of the SAP gels, highlighting the influence of particle size and molecular chain order on extended-release dynamics.

#### The Effect of Temperature on the Water Retention of the SAPs

**Figure 12:**
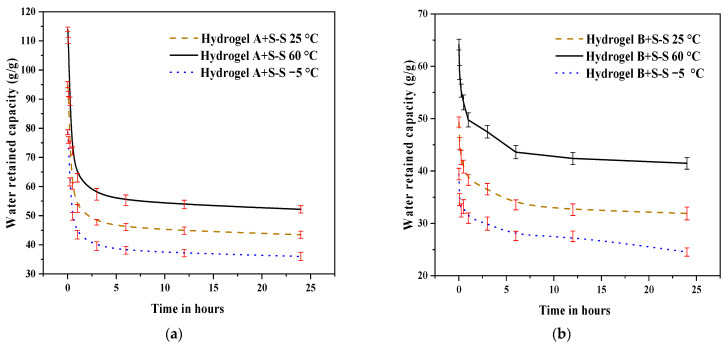
Effect of water retention capacity of SAP hydrogels with S-S subjected to different temperature within 24 h for: (**a**) SAP-a, and (**b**) SAP-b.

Figure 12 presents the water release and retention capacities of different SAPs (SAP-a, SAP-b, SAP-c, and SAP-d) under different temperatures (−5 °C, 25 °C, 60 °C) within 24 h time intervals. We conducted an experiment to simulate SAP behavior in cement paste with the addition of sodium silicate (SS). Under cold conditions (−5 °C), the water release capacities were remarkably reduced for SAP-a, exhibiting 65.3 g/g, representing a reduction of approximately 33.7% compared to 25 °C. While at 25 °C, the water release capacities exhibited by SAP-a, which retained the highest amount of water, were 82.7 g/g to 229 g/g. Conversely, at 60 °C, a substantial increase in water release was achieved; for instance, SAP-a released 113.98 g/g, reflecting an improvement of around 20.9% compared to 25 °C. The hierarchical order of SAP types, based on particle size and molecular chain length (SAP-a > SAP-b > SAP-c > SAP-d), correlates with their respective water retention capacities at 25 °C. 

### 3.4. Fluidity of SAP Mortars

To study the flowability of the SAP mortars, two SAP types, SAP-a and SAP-d, were selected among the four types of SAPs used in this study. The selection of SAP-a and SAP-d was based the molecular chains and particle sizes of these SAPs, with SAP-a having the largest molecular chain and particle size, meaning it absorbed more water compared to the other SAP types, and SAP-d having the smallest molecular chain and particle size, along with the lowest water absorption capacity.

**Figure 13:**
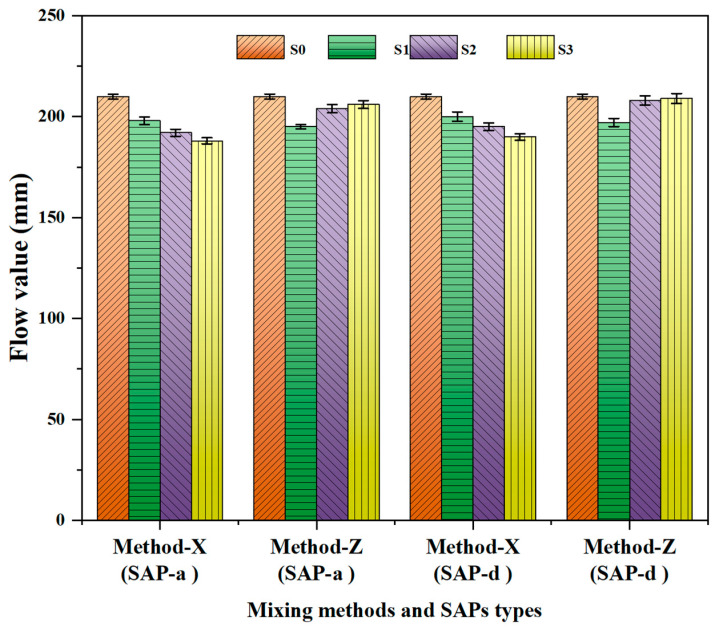
The flowability of mortars containing SAP-a and SAP-d. S0 represents the control group; S1, S2, and S3 refer to the SAP-modified mortar mixes containing 0.1%, 0.2%, and 0.3% by cement weight, respectively.

The Influence of the SAPs on the fresh properties of the cement mortars is shown in Figure 13. Introducing dry SAPs with varying SAP dosages led to a significant decrease in slump. Conversely, the mortar mixtures containing pre-wetted SAPs exhibited an 11.9%, 7.1%, and 3.33% increase in slump compared to the reference mixture at SAP dosages of 0.1%, 0.2%, and 0.3%, respectively. The SAP mortar prepared under Method-X showed decreases in the slump value for the types of SAPs (SAP-a and SAP-d) utilized for the flow test. Conversely, the flowability of SAP mortars prepared with dry SAPs decreased first and then increased with SAP content (Method-Z was used to prepare SAP-a and SAP-d), as shown in Figure 13. This behavior is attributed to the effect of dry SAPs on fresh mortar density, leading to increased yield stress and plastic viscosity. Dry SAPs absorb water from the fresh mix, enhancing the density of the mixture and generating air voids. Including dry SAPs may necessitate an increased dosage of superplasticizer to maintain good workability. Conversely, for the SAP mortars constructed using the pre-wetted SAP mixing procedure, the flow value increased significantly with SAP dosage. After mixing, the internally cured mortars released water into the cement paste, increasing the mortar mixture’s flowability. Reduced internal humidity has a great effect on the SAP release in mortars.

### 3.5. Mechanical Properties of SAP Mortars

#### 3.5.1. Compressive Strength

**Figure 14:**
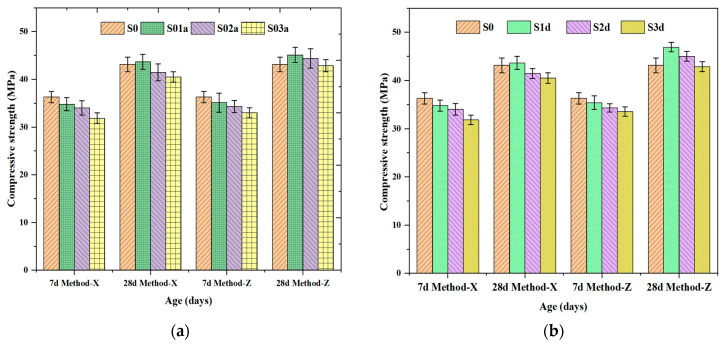
Effect of SAPs on compressive strength of mortars. (**a**) SAP-a and (**b**) SAP-d. S0 represents the control group; S01a, S02a, and S03a refer to SAP-a-modified mixtures with 0.1%, 0.2%, and 0.3% by cement weight, respectively; S01d, S02d, and S03d signify SAP-d-modified mixtures containing 0.1%, 0.2%, and 0.3% by cement weight, respectively.

The influence of SAP particle size, mixing procedure, and superabsorbent polymer (SAP) dosages on the 7- and 28-day compressive strength of mortar specimens was also evaluated, as shown in Figure 14. The main factors that affected the compressive strength of the mortars were the SAP types and dosages added to the mixture. A notable decline in compressive strength was observed with SAP content. Specifically, the 7 d strength reduction for specimens S01a-X, S02a-X, and S03a-X, which were in the dry mixing method group, was 4.11%, 6.28%, and 12.2%, respectively, compared to the reference specimen. In the pre-wetting mixing method group, mixtures S01a-Z, S02a-Z, and S03a-Z exhibited reductions of 3.3%, 5.5%, and 9.1%, respectively, compared to the control mixture. Conversely, mixtures S01d-X, S02d-X, S03d-X, S01d-Z, S02d-Z, and S03d-Z demonstrated reductions of only 5.5%, 8.1%, 10.7%, 2.5%, 5.4%, and 7.6%, respectively, relative to the reference specimen, which possessed a 7-day compressive strength of 36.3 Mpa. The decrease in strength at the early stage is attributed to macro-pores formed due to water release by SAPs during hydration [11]. Interestingly, a marginal increase in compressive strength was noted upon adding SAPs at 0.1 wt.% and 0.2 wt.% in the pre-wetting method (Method-Z), as depicted in Figure 14a,b. For instance, mixtures S01a-Z, S02a-Z, S01d-Z, and S02d-Z exhibited increases of 4.6%, 2.9%, 8.8%, and 4.3%, respectively, in 28-day compressive strength compared to the reference specimen. Generally, the compressive strength was reduced, and the pre-wetting method achieved greater compressive strength than the dry mixing method. This decrease is related to the micro-voids generated by the larger SAP particles, which release a greater amount of water in their network than SAP-d upon completing hydration [36].

#### 3.5.2. Flexural Strength 

**Figure 15:**
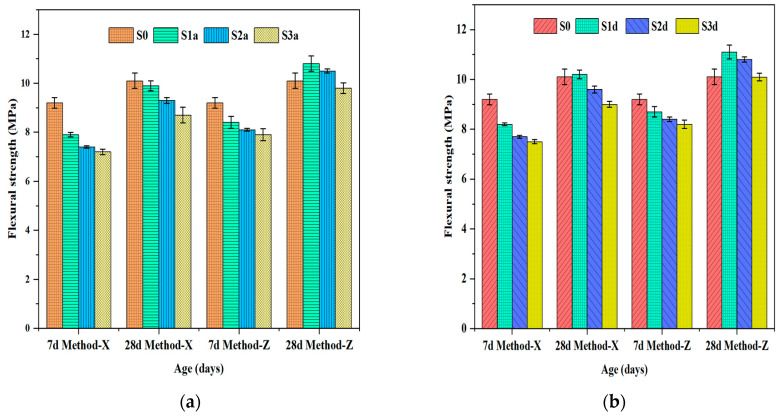
Flexural strength of SAP mortars: (**a**) SAP-a; (**b**) SAP-d. S0 represents the control group; S01a, S02a, and S03a refer to SAP-a-modified mixtures with 0.1%, 0.2%, and 0.3% by cement weight, respectively; S01d, S02d, and S03d signify SAP-d-modified mixtures containing 0.1%, 0.2%, and 0.3% by cement weight, respectively.

Figure 15 shows the flexural strength test results of the SAP mortars prepared with different SAP contents using the dry (Method-X) and pre-wetting (Method-Z) methods. Similarly, the SAP mortars revealed decreases in flexural strength with increased SAP content. The mortar mixtures S01a-X, S02a-X, and S03a-X, shown in Figure 15a, decreased by 14.13%, 19.57%, and 21.74% at 7d of curing age, respectively. Similarly, the SAP mortars derived from the pre-wetting method (Method-Z) showed a reduction in flexural strength. The flexural strength decreased by 8.7%, 11.96%, and 14.13% compared to the control specimen. The reduced flexural strength is attributed to a poor bond between the SAP particles and the cement paste. Adding 0.1% SAP resulted in the highest flexural strength in the pre-wetted mixtures. The flexural strength of mixes S01a-Z and S01d-Z rose by 7% and 10%, respectively, as compared to the reference specimens. At later ages, the flexural strength of the pre-wetted SAPs was higher than that of the reference, but it decreased with the increase in SAP dosage [37]. 

### 3.6. Autogenous Shrinkage Properties

**Figure 16:**
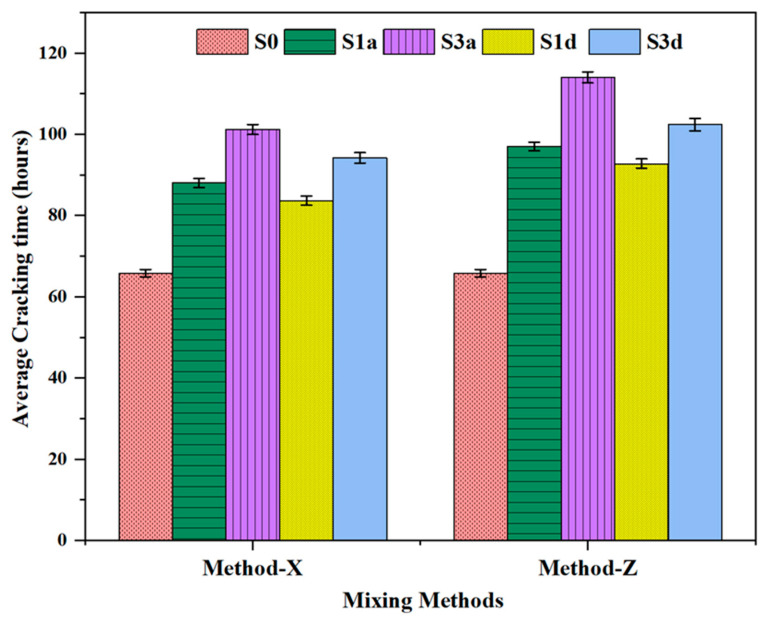
Shrinkage properties of SAP mortars: S0 represents the control group; S01a, S02a, and S03a refer to SAP-a-modified mixtures with 0.1%, 0.2%, and 0.3% by cement weight, respectively; S01d, S02d, and S03d signify SAP-d-modified mixtures containing 0.1%, 0.2%, and 0.3% by cement weight, respectively.

Figure 16 presents the cracking strength values of SAP mortars based on two types of SAPs (SAP-a and SAP-d). In general, including these SAPs resulted in a delay in the onset of mortar cracking, which improved with SAP content for the two mixing procedures. According to the SAP content, the mortar containing SAP-a exhibited a cracking time more significant than that of the reference specimen by 1.27 times, 1.43 times, 1.41 times, and 1.56 times. Similarly, the mortar prepared with SAP-d exhibited cracking times that were higher by 1.33 times, 1.54 times, 1.47 times, and 1.73 times. SAP-a exhibited a greater water absorption capacity. Still, its large particle size may lead to rapid water release, inadequate for the stabilization of the reduction in the internal humidity of the specimen. Incorporating 0.3 wt.% pre-wetted SAP-d resulted in a significant cracking strength despite its lower water retention capacity. The average cracking time in specimens with SAP-d was 4 to 8 h. This discrepancy can be attributed to the controlled release of SAP-d with a short molecular chain, enabling it to gradually absorb mixing water and gradually release it to the cement paste. This, in turn, mitigates the reduction in moisture gradients in the concrete during later stages, facilitating higher expansion during the hydration process [34].

### 3.7. ANOVA Results

A three-way ANOVA was adopted in this study to clearly understand the influence of various independent variables on the key properties of SAP mortars, including fluidity, compressive strength, flexural strength, and shrinkage, as summarized in Table 5, Table 6, Table 7 and Table 8, respectively. Our comprehensive analysis employing a three-way ANOVA approach elucidated the intricate effects of various variables, such as SAP dosage, SAP type, and mixing method, on the fluidity, mechanical strength, and shrinkage properties of cement mortars modified with SAPs. The fluidity tests indicated that while SAP type and mixing method did not significantly affect the flow values, the dosage of SAP emerged as a critical factor, suggesting a nuanced interplay between SAP concentration and the workability of mortar mixtures. Additionally, the interaction effects between these variables were found to be generally nonsignificant, indicating that each variable independently influences the mortar’s fluidity to varying degrees. In contrast, the mechanical strength of the cement mortars, assessed through compressive and flexural strength tests, revealed a pronounced sensitivity to all tested variables, particularly the curing time, which showed highly significant *p*-values across both strength measures. This underscores the curing process’ critical role in developing the mechanical properties of SAP-modified cement mortars. Similarly, the investigation into mortar shrinkage through the metric of average cracking time further highlighted the paramount importance of SAP dosage, with a remarkably low *p*-value indicating a strong correlation between SAP dosage and the mitigation of shrinkage-induced cracking.

## 4. Conclusions

This study investigated the water retention capacities of SAPs in an alkaline environment over extended periods by incorporating liquid sodium silicate (SS) into SAP–water mixtures and examining the influence of temperature. The effect of SAP particle size on the water absorption capacities and mechanical mortars was investigated. Two mixing techniques for SAPs (dry and pre-wetting) were employed to assess the influence of SAPs on cement mortars’ slump, mechanical properties, and cracking resistance. Furthermore, a three-way analysis of variance (ANOVA) was conducted to understand the influence of different independent variables on the fluidity, mechanical strength, and shrinkage properties of SAP-modified cement mortars. The key findings are summarized below:

1. The SAPs exhibited rapid water absorption, exceeding 85% within the first 30 min before reaching a saturation point. This equilibrium state occurs when the osmotic pressure within the gel and the surrounding environment equalizes. The chemical and temperature variations in the water significantly affect water absorption and desorption. However, the SAP hydrogel retains a portion of water, even in an alkaline environment, for extended periods unless there is a significant change in pH or ion concentration.
2. The simulations conducted in an alkaline environment created by adding SS indicate that the ion concentration remains constant. In contrast, in a cementitious climate, the ion concentration increases with time (hydration degree) as the SAP releases water over days.
3. The filtration experiments revealed that SAP-d exhibited the slowest water release rate, retaining water for significantly longer than the other three types, making it particularly beneficial for delaying cracking and reducing shrinkage. The addition of SAP content using the dry mixing method reduces mortar fluidity. Conversely, pre-wet blending with SAPs results in a slight increase in fluidity as the SAP content increases.
4. The compressive and flexural strength of SAP mortars is reduced due to the addition of SAPs at early ages. Under the dry mixing method, compressive and flexural strength decreases with increases in SAP content due to the formation of larger macro-voids. However, pre-wet mixing leads to increased strength at later ages compared to dry-mixing, attributed to the enhanced hydration reaction triggered by the water released from the reservoirs.
5. SAP incorporation delays the average cracking time of mortars with increasing dosage. Superabsorbent polymers effectively control shrinkage stability and prevent premature cracking when the dosage reaches 0.3%. SAP-d, with its slow release and smaller bulk surface, also demonstrated a superior ability to inhibit the early cracking of cement mortars.
6. The statistical analyses provided clear evidence of the significant impact of SAP dosage, curing time, and mixing method on the fluidity, mechanical strength, and shrinkage characteristics of cement mortars. These findings underscore the critical importance of optimizing SAP modifications to enhance the performance and durability of construction materials.

## Figures and Tables

**Table 1:** Cement chemical composition.

Cement	Oxides						
OPC	SiO_2_	Al_2_O_3_	Fe_2_O_3_	CaO	MgO	SO_3_	LOI
	21.20	4.64	3.13	61.24	4.60	1.76	2.15

**Table 2:** Physical and water absorption characteristics of SAPs.

Properties	SAP-A	SAP-B	SAP-C	SAP-D
Appearance	White	White	White	White
Fineness (mesh)	30–60	60–100	120–180	300–400
Absorption in tap water/(g/g)	≥200	≥130	≥100	≥90
Water holding capacity (0.9% NaCl)/(g/g)	≥60	≥60	≥50	≥45
Bulk density g/m	0.65–0.85	0.65–0.85	0.65–0.85	0.65–0.85
pH values	5.5–6.5	5.5–6.5	5.5–6.5	5.5–6.5

**Table 3:** Chemical composition of sodium silicate.

Na_2_O (%)	SiO_2_ (%)	Baume Degree (20 °C)	Solid Content (%)	pH Value
≥12.80	≥29.20	50.0–51.0	42.0	14.0

**Table 4:** Mix proportion (unit: kg/m^3^).

Materials	Method-X				Method-Z		
	0	0.1%	0.2%	0.3%	0.1%	0.2%	0.3%
Cement	530	530	530	530	530	530	530
Water	223	223	223	223	223	223	223
Fine aggregate	1325	1325	1 325	1325	1325	1325	1325
SAP	-	0.53	1.06	1.59	0.53	1.06	1.59
Water-reducing agent	2.1	2.1	2.1	2.1	2.1	2.4	2.4

**Table 5:** Summary of fluidity test statistics via three-way ANOVA.

Flow Value (mm)						
Variables	A: SAP type	B: SAP dosage	C: Mixing method	Interaction AB	Interaction BC	Interaction AC
*p*-values	0.545629	0.007302	1.000	0.133070	0.177954	0.648050

**Table 6:** Impact of different variables on mechanical strength of cement mortars modified with SAP-a (determined via three-way ANOVA).

Variables	*p*-Values	
	Compressive Strength (MPa)	Flexural Strength (MPa)
A: SAP dosage	0.015711	0.001812
B: Curing time	0.000069	0.000071
C: Mixing method	0.029501	0.001411
Interaction AB	0.113222	0.009991
Interaction AC	0.264351	0.024546
Interaction BC	0.103837	0.060931

**Table 7:** Summary of statistics on the influence of different variables on mechanical strength of cement mortars modified with SAP-d. Statistics were determined using three-way ANOVA.

Variables	*p*-Values	
	Compressive Strength (MPa)	Flexural Strength (MPa)
A: SAP dosage	0.035844	0.003410
B: Curing time	0.000186	0.000071
C: Mixing method	0.031857	0.001411
Interaction AB	0.175931	0.009991
Interaction AC	0.351723	0.024546
Interaction BC	0.122590	0.060931

**Table 8:** Effects of variables on cement mortar shrinkage–average cracking time.

Average Cracking Time (h)					
Variables	A: SAP dosage	B: SAP type	C: Mixing method	Interaction BC	Interaction AC
*p*-values	0.000384	0.075096	1.000	0.625455	0.114590

## Data Availability

Data is contained within the article.
